# Risk Factors Associated with Attempted Suicide  : A Case Control Study

**Published:** 2004

**Authors:** M. K. Srivastava, R N Sahoo, L H Ghotekar, Srihari Dutta, M. Danabalan, T K Dutta, A K Das

**Affiliations:** 1 Chief Medical Officer, Department of Community Medicine, Jawaharlal Institute of Postgraduate Medical Education and Research (JIPMER) Pondicherry 605006, India; 2 Department of Forensic Medicine, Jawaharlal Institute of Postgraduate Medical Education and Research (JIPMER) Pondicherry 605006, India; 3 Department of Medicine, Jawaharlal Institute of Postgraduate Medical Education and Research (JIPMER) Pondicherry 605006, India; 4 Senior Resident, Department of Community Medicine, Jawaharlal Institute of Postgraduate Medical Education and Research (JIPMER) Pondicherry 605006, India; 5 Professor and Head, Department of Community Medicine, Jawaharlal Institute of Postgraduate Medical Education and Research (JIPMER) Pondicherry 605006, India; 6 Professor, Department of Medicine, Jawaharlal Institute of Postgraduate Medical Education and Research (JIPMER) Pondicherry 605006, India; 7 Dean, Director Professor & Head Department of Medicine, Jawaharlal Institute of Postgraduate Medical Education and Research (JIPMER) Pondicherry 605006, India

**Keywords:** Attempted suicide, Risk factors, Odds ratio, Pain

## Abstract

This study was conducted to identify the risk factors associated with attempted suicide among people living in and around Pondicherry. Using a case control study design, 137 consecutive cases of attempted suicide admitted to Jawaharlal Institute of Postgraduate Medical Education and Research, a teaching hospital in Pondicherry and an equal number of controls, matched individually with cases for age and sex, from the relatives and friends of the other patients, were studied. Variables related to socio demographic characteristics, family background, recent stressful life events, physical and psychiatric morbidity were analysed. The strength of association with the risk of attempt was calculated using odds ratio with 95% confidence interval. Odds ratios for the factors identified to be significantly associated with increased risk of suicide attempt were 15.82(95% CI 6.55-40) for unemployment, 3.02 (95%CI 1.78-5.14) for lack of formal education, 3.95 (95% CI 2.02-7.79) for the presence of stressful life events in the last six months, 3.12 (95%CI 1.37-7.24) for suffering from physical disorders and 6.78 (95% CI 2.39-2070) for suffering from idiopathic pain. Significant association was not revealed in respect to marital status, type of family, early parental losses, family history of suicide and presence of psychiatric morbidity.


ORIGINAL ARTICLE

*Indian Journal of Psychiatry, 2004, 46(I)33-38*

### Risk Factors Associated with Attempted Suicide : A Case Control Study

**M.K. Srivastava*[1] R.N. Sahoo[2] L.H. Ghotekar[3] SrihariDutta[4] M. Danabalan[5] T.K. Dutta[6] A.K. Das[7]**

**Abstract**

This study was conducted to identify the risk factors associated with attempted suicide among people living in and around Pondicherry. Using a case control study design, 137 consecutive cases of attempted suicide admitted to Jawaharlal Institute of Postgraduate Medical Education and Research, a teaching hospital in Pondicherry and an equal number of controls, matched individually with cases for age and sex, from the relatives and friends of the other patients, were studied. Variables related to socio demographic characteristics, family background, recent stressful life events, physical and psychiatric morbidity were analysed. The strength of association with the risk of attempt was calculated using odds ratio with 95% confidence interval. Odds ratios for the factors identified to be significantly associated with increased risk of suicide attempt were 15.82(95% CI 6.55-40) for unemployment, 3.02 (95%CI 1.78-5.14) for lack of formal education, 3.95 (95% CI 2.02-7.79) for the presence of stressful life events in the last six months, 3.12 (95%CI 1.37-7.24) for suffering from physical disorders and 6.78 (95% CI 2.39-2070) for suffering from idiopathic pain. Significant association was not revealed in respect to marital status, type of family, early parental losses, family history of suicide and presence of psychiatric morbidity.

Key Words: Attempted suicide, Risk factors, Odds ratio, Pain,

### INTRODUCTION

Attempted suicide is stated to be associated with several psychosocial and medical conditions. Young age of 15 to 24 years, female sex, poor education, unemployment, living alone and history of socioeconomic deprivation are stated to be potential risk factors (Schmidtke et al., 1996). International studies have also identified a role of adverse family factors such as parental loss in childhood, family discordance psychiatric antecedents and exposure of suicide in family (Chastang et al., 1998; Botsis et al, 1995). Attempters were reported to have experienced more number of stressful life events in comparison to general population and precipitating events were found to be often interpersonal problems (Paykel et al., 1974; Hawton et. al., 1982). Physical and psychiatric illnesses are also reported to play important roles (Miles, 1977; Runeson, 1989). However studies have failed to reveal consistent findings with regard to the prevalence and potency of these risk factors in different regions. (Kirmayer et al., 1996). Human suicide is a complex phenomenon. At times it appears to be a very private affair, not even shared by the closest ones; on the other hand, wide variations in suicide rates across different regions signify a predominant role of cultural factors. The culture of our society is markedly different from that of the west. There is need to identify the factors associated with the suicide attempt pertaining to our cultural norms. It is not known whether the risk factors reported in studies from western countries are universal and also common among the population of our country. The present study aims to identify risk factors associated with suicide attempt with reference to socio-demographic characteristics, family background, stressful life events, physical and psychiatric morbidity in the population living in and around Pondicherry.

### Materials and Methods:

This study was conducted at Jawaharlal Institute of Postgraduate Medical Education and Research Hospital. This is a multi-specialty teaching hospital in the Union Territory of Pondicherry and caters mainly to people residing in Pondicherry and adjoining areas of Tamil Nadu. For the purpose of present study, a case of attempted suicide was defined as " A person who had made deliberate act of self harm consciously aimed at self destruction,

[1]Chief Medical Officer, Department of Community Medicine,
[2]Department of Forensic Medicine
[3]Department of Medicine
[4]Senior Resident, Department of Community Medicine
[5]Professor and Head, Department of Community Medicine
[6]Professor, Department of Medicine
[7]Dean, Director Professor & Head Department of Medicine
Jawaharlal Institute of Postgraduate Medical Education and Research (JIPMER) Pondicherry 605006
*Correspondence: Dr. M.K. Srivastava, DII-8, JIPMER Campus, Pondicherry 605 006 Email address manoharkant@yahoo.co.in irrespective of his or her intention to die, with non fatal outcome". Based on this criteria 137 consecutive cases of suicide attempt admitted to the Medical Wards over a period of 4 months (July99 to October 99) were identified and included for the study. Cases with burn injuries were not included.

Each case was interviewed shortly after his/her medical condition became stable. Information related to their socio-demographic characteristics, detailed account of present attempt and past attempt if any, history of past illnesses, personal history, family type, history of illnesses, suicide/ suicide attempt in family and parental loss before the age of 12 years were recorded in a pre-designed proforma. The presumptive stressful life events scale (Singh et al., 1983) was administered to assess stressful life events experienced during the 6 months prior to suicide attempt. Each individual was also subjected to a detailed physical and psychiatric examination including relevant investigations and the respective diagnoses were recorded as per the criteria of International Statistical Classification of Diseases and Related Health Problems, 10th revision (WHO 1992) and ICD 10 Classification of Mental and Behavioural Disorders (WHO, 1992).

An equal number of controls, "who had never made suicide attempt", individually matched for each case in respect to age (± 2 years) and sex were recruited from the relatives and friends of other patients of same geographical area admitted in the wards during that period. They were also subjected to similar procedures of interview and clinical examinations after obtaining an informed consent from each of them.

Statistical analyses included calculation of means, frequencies and percentages of the variables as descriptive measures. The results were analysed using Chi Square test and also appropriate Fisher's exact test to find any significant difference. Each variable was collapsed into dichotomous form and the strength of association with the risk of attempt was calculated as odds ratio with 95% confidence interval using computerized statistical package Epi Info (2000) 1.0 version.

### RESULTS

Table 1 shows the age and sex distribution of cases studied by us. Mean age was 25.31 ( SD=9.04 ) years and majority of them were young. With regard to sex 65 were males and 72 females. Table 2 illustrates the sociodemographic characteristics of the cases and the control subjects in our study. Among cases majority 77(56.2%) were married 105(76.6%) from nuclear families, 76(55.5%) uneducated and 63(46%) were unemployed. Among the control subjects, who were matched for age and sex with the cases, 70(51%) were married, 109(79.5%) from nuclear families, 40(29.8%) uneducated and only 7(5.1%) were unemployed.

Table 1

**Age and sex distribution of cases of attempted suicide**

| Age group (in years) | Males (n=65) | Females (n=72) | Total (n=137) |
|---|---|---|---|
| 14-19 | 9 (13.8%) | 32 (44.5%) | 41(29.9%) |
| 20-24 | 17 (26.2%) | 12 (16.7%) | 29 (21.2%) |
| 25-29 | 20 (30.8%) | 15 (20.8%) | 35 (25.5%) |
| 30-34 | 6 (9.2%) | 6 (8.3%) | 12 (8.8%) |
| 35-39 | 6 (9.2%) | 5 (6.9%) | 11(8.03%) |
| 40 and above | 7 (10.8%) | 2 (2.8%) | 9 (6.5%) |

Table 2

**Socio-demographic characteristics of cases and controls**

| ***Variables*** | ***Cases*** *(n=137)* | ***Controls*** *(n=137)* |
|---|---|---|
| *Marital status* | | |
| *Married* | *77 (56.2%)* | *70 (51.1%)* |
| *Unmarried* | *60 (43.8%)* | *67 (48.9%)* |
| *Type of family* | | |
| *Nuclear* | *105 (76.6%)* | *109 (79.5%)* |
| *Joint* | *32 (23.4%)* | *28 (20.5%)* |
| *Educational Status* | | |
| *No formal education* | *76 (55.5%)* | *40 (29.8%)* |
| *Studied up to* | | |
| *Primary* | *32 (23.3%)* | *48 (35.1%)* |
| *Secondary* | *27(19.7%)* | *39 (28.5%)* |
| *Higher secondary* | *2 (1.5%)* | *10.(7.2%)* |
| *Occupational status* | | |
| *Unemployed* | *63 (46%)* | *7 (5.1%)* |
| *Employed as* | | |
| *Farmer* | *21 (15.3%)* | *33 (24.1%)* |
| *Labourer* | *36 (26.3%)* | *44 (32.1%)* |
| *Driver* | *2 (1.5%)* | *8 (5.8%)* |
| *Housewife* | *10 (7.3%)* | *25 (18.2%)* |
| *Student* | *5(3.6%)* | *20(14.5%)* |

Table 3 shows the family history, past history of suicide, parental loss and recent stressful life events in the cases and controls. In 6(4.34%) cases there was history of parental loss, 2(1.5%) cases had history of suicide /suicide attempt in their family, and 2 (1.5%) cases had history of pervious suicide attempt. In the controls, parental loss was found in only one subject and none had any history of suicide in their family. Forty-seven(34.3%) cases and 16(11.75) control subjects had experienced stressful life events during previous six months. These events were mostly of undesirable types and were related to the interpersonal conflicts, financial problems like loan and unemployment.

Table 3

***Life events and family factors in cases and controls***

| Events /Family factors | Cases (n=137) | Controls (n=137) |
|---|---|---|
| *Early parental loss* | | |
| *Death of mother* | *3(2.2%)* | *0* |
| *Death of father* | *1(0.73%)* | *1* |
| *Parental divorce* | *2(1.5%)* | *0* |
| *Total* | *6(4.34%)* | *1(0.73%)* |
| *Previous suicide attempt* | *2 (1.5%)* | *0* |
| *Family history of suicide* | | |
| *Suicide attempt* | *1(0.73%)* | |
| *Died of suicide* | *1(0.73%)* | |
| *Total* | *2 (1.5%)* | *0* |
| *Life events in last six months prior to suicide attempt* | | |
| *Undesirable* | | |
| *Related to financial problems* | *24(17.5%)* | *12(8.8%)* |
| *(Losses, loan, unemployment)* | | |
| *Related to interpersonal conflicts (Marital and family conflict)* | *11(8.03%)* | *2(1.5%)* |
| *Others (major personal illness, death of family member, lack of child)* | *10(7.3%)* | *1(0.73%)* |
| *Desirable* | *2(1.5%)* | *1(0.73%)* |
| *Marriage* | | |
| *Total* | *47(34.3%)* | *16(11.7%)* |

Self-poisoning was the commonest method adopted for attempting suicide. This was used by 137 cases while 5 cases had made attempt by hanging. Problems like arguments with family members, quarrels with the spouse, financial problems related to unemployment and difficulty in the repayment of the loan were the reason of attempting suicide found in 82(57.8%) cases whereas 52(37.5%) cases stated the reason being their sufferings from chronic pain and illnesses.

Table 4 shows the diagnostic distribution of the associated illness in the cases and controls subjects. Among the cases 16(11.6%) had psychiatric disorders. Alcohol dependence was the commonest, found in 11 of the 16 cases and two received the diagnosis of depressive disorder. Others were identified to be of schizophrenia, personality disorder, and conduct disorder. In 27(19.7%) cases physical illnesses were identified. Among these dysmenoherrea was the commonest found in 15 cases and peptic ulcer disease in 9 cases. In the rest, one case each were of hypertension, bronchial asthma and arthritis. In twenty-eight cases (20.44%) there was no diagnosable illness. Pain was the only symptom, which was found to be vaguely localized in

Table 4

**Clinical characteristics of cases and controls: Diagnosis as per ICD -10 criteria**

| Diagnosis | Cases (n=137) | Controls ( n=137) |
|---|---|---|
| Psychiatric disorders | | |
| Alcoholism | 11 (8.03%) | 8 (5.84%) |
| Depressive disorder | 2 (1.46%) | 0 |
| Schizophrenia | 1 (0.73%) | 0 |
| Personality disorder | 1 (0.73%) | 0 |
| Conduct disorder | 1 (.0.73%) | 0 |
| Total | 16 (11.7%) | 8 (5.84%) |
| *Physical disorder* | | |
| *Dysmenorrhea* | *15 (10.95%)* | *4 (2.92%)* |
| *Peptic ulcer* | *9 (6.57%)* | *1 (0.73%)* |
| *Bronchial asthma* | *1 (0.73%)* | *2 (1.46%)* |
| *Arthritis* | *1 (0.73%)* | *1 (0.73%)* |
| *Hypertension* | *1 (0.73%)* | *1 (0.73%)* |
| *Diabetes* | *0* | *1 (0.73%)* |
| *Total* | *27 (19.71%)* | *10 (7.23%)* |
| *Symptoms without associated illness* | | |
| *Abdominal & pelvic pain* | *28 (20.44%)* | *5 (13.65%)* |
| *All diagnosis* | *71 (51.8%)* | *23 (16.79%)* |

the abdominal and pelvic region and was without any associated illness. This idiopathic pain on clinical assessment fulfilled the criteria of " abdominal and pelvic pain" and a diagnosis of "symptoms, signs and abnormal and laboratory findings not elsewhere classified" as per International Statistical Classification of Diseases and Related Health Problems 10th revision (WHO, 1992) was made. Among the controls, 8 had psychiatric disorder, 10 physical illness and 5 had the symptom of idiopathic pain similar to the cases.

Table 5 shows comparison of variables between cases and controls. All variables are expressed in dichotomous form and the strength of association with suicide attempt for the identification of risk factors is denoted by unadjusted odds ratio (OR) with 95% confidence interval (CI).

With respect to the sociodemographic variables, those who were unemployed and uneducated were found to be at a significantly higher risk for attempting suicide. Odds ratios were 15.81(95%CI 6.55-40, P<0.0001) for being unemployed and 3.02 (95% CI 1.78-5.14,P<0.0001) for lack of formal education. Marital status and type of family were not found to be associated significantly.

A statistically significant association was found with recent

Table 5

**Comparison of Socio-Demographic variables, Life events, Family factors and Clinical variables between cases and controls (each variable separately)**

| | Cases (n=137) | Controls (n=137) | Unadjusted odds ratio (95% Confidence interval) |
|---|---|---|---|
| Demographic variables | | | |
| Unmarried | 60(43.8%) | 67(48.9%) | 0.81 (0.49-1.35) |
| Living in nuclear family | 105(76.6%) | 109(79.6%) | 0.84 (0.46-1.55) |
| Uneducated | 76(55.5%) | 40(29.2%) | 3.02** (1.78-5.14) |
| Unemployed | 63(45.9%) | 7(5.1%) | 15.81** (6.55-40) |
| Events & family factors | | | |
| At least one event in last 6 months | 47(34.3%) | 16(11.7%) | 3.95** (2.02-7.79) |
| Family history of suicide | 2 (1.5%) | 0 | *** —- |
| Previous suicide attempt | 2(1.5%) | 0 | *** —- |
| Early parental loss | 6(4.3%) | 1(0.73%) | 6.23 (0.73-139-16) |
| Clinical variables: | | | |
| Physical disorders | 27(19.78%) | 10(7.29%) | 3.12* (1.37-7.24) |
| Psychiatric disorders | 16(11.67%) | 8(5.84%) | 2.13 (0.82-5.66) |
| Disorders not classifiable elsewhere | 28(20.44%) | 5(3.6%) | 6.78** (2.39-20.76) |

*P<0.001, **P<0.0001
*** Odds ratio could not be computed because these variables were not present in the controls.

stressful life event. Those who had experienced life events in the previous six months were found to be at higher risk. (OR 3.95; 95% CI 2.02-7.79, p<0.0001). Family history of suicide and parental loss before the age of 12 years were not found to be significantly associated. Odds ratio in respect to these variables could not be calculated, as these variables were not present in the controls.

With respect to the associated illnesses a significant association was found with pain and physical illnesses. Cases suffering from idiopathic pain and physical illnesses were found to have a significantly increased risk in comparison to the controls. Odds ratios were 3.12 (95% CI 1.37-7.24,P<0.001) for physical disorders and 6.78 (95% CI 2.39-20.76, P<0.0001) for suffering with idiopathic pain. Further, association with respect to the psychiatric illness was not found to be statistically significant.

### DISCUSSION

The present study is an attempt to find out risk factors associated with suicide attempt. The sample in our study consisted of cases admitted in the hospital and the controls from the relatives and friends of other patients admitted during the same period for the treatment of other ailments matched individually with cases for the age and sex. We found majority (51%) of cases were young, below 24years and there was female predominance. This finding is in agreement with other studies (Schmidtke, et. al., 1996, Sathyavathi, 1971;Badrinarayana, 1977;Ponnudurai, 1986; Narang, et. al., 2000). Only 6.5% cases were of 40years and above. Older people have better coping skills and more likely to use cognitive appraisal to manage stress in comparison to the young (Folkman et al, 1987). We feel that this could be one of the reasons for low prevalence of suicide attempt among old aged people.

No significant difference between the cases and the controls was found in our study with respect to the marital status or the type of family. People living alone were reported to be at higher risk in Western studies (Schmidtke et. al, 1996) where as reports of Indian studies on this aspect is conflicting. Narang et al. (2000) found suicide attempt being more common in the unmarried while Lal and Sethi (1975) reported it to be more common in married persons. It implies that risk may be hidden in the interpersonal relations of the members of family living together rather than their family type and marital status.

Unemployment was identified as the strongest predictor. Odds of making attempt were found to be 15.81 times higher among the unemployed compared to the employed (OR 15.81 95% CI 6.55-40). Further odds of making attempt were 3.02 times higher among uneducated compared to the educated (OR 3.02, CI 1.78-5.14). This is in agreement with findings of other researches (Schmidtke et al., 1996).

Further in agreement with other researches (Chastang et al., 1998; Paykel, 1974; Latha, et al., 1996 and SudhirKumar et al., 2000) exposure to stressful life events in recent past was also found to be another significant risk factor associated with suicide attempt. (OR 3.02 95% CI 2.02-7.79). So far the types of events concerned in majority of cases the events were related to financial problems. Hintikka et.al.(1998) also reported debt and experiencing financial difficulties in repayment of loan as a factor associated independently with suicide attempt. These findings reveal that there is need to explore further for a link between factors related between the lack of education and unemployment and the financial problems.

An important finding of this study is pain and physical disorders being identified as a significant risk factor associated with suicide attempt. On comparison with controls, odds ratios were 3.12(95% CI 1.37-7.24, P<0.001) with regard to physical disorders and 6.78(95% CI2.39-20.76, P<0.0001) with regard to pain. In our study pain in most of the cases was found to be localized in abdominal and pelvic region It was idiopathic in 28(20.4%) cases and associated with disorders like dysmenohrrea and peptic ulcer disease in 24 out of 27 cases of physical illness. This idiopathic pain fulfilled the diagnostic criteria of "abdominal and pelvic pain" under the diagnostic category of "symptoms, signs and abnormal clinical and laboratory findings not classifiable elsewhere" of International Statistical Classification of Diseases and Related Health Problems 10th revision (WHO, 1992). This finding is consistent with the observation of Sathyavathy and Murti Rao (1961) who reported stomachache in 86 out of 261 cases in their study at Bangalore. Abdominal pain, both functional and associated with disorders like dysmenorrhea and with peptic ulcer was also reported to be present in suicide attempters by Venkoba Rao (1971) from Madurai. Very few studies had attempted to explore the relation between the physical disorder and suicide. Prevalence of physical disorder among attempters is stated to vary from 20 to 50%. And 13 to 21% patients in such studies had pain with variable presentations like headache, joint pain, muscle pain, lumbar pain, and gastric pain, (Stenager, 2000). This finding merits attention. Jensen et al (1991) pointed out that studies had shown that majorities of chronic pain patients are not clinically depressed and many of them do not seek medical advice and continue to work despite experiencing pain frequently. They advocated that it is important to identify the factors, which promote adaptive functioning. In their literature review they had concluded that a complex relationship exists among pain appraisal, coping strategy and adjustment to pain. Further studies are needed to identify these factors.

Another finding with regard to the associated illness is that psychiatric illnesses were not associated significantly with the suicide attempt. A low prevalence, 11.6% (16 out of 137) psychiatric disorders was found in cases studied by us which is not in the agreement with the several studies (Souminen et al, 1996, Sudhirkumar et. al. 2000, Narang et. al. 2000). But in our study out of 137 cases, except 2 all were first attempters and our findings are in agreement with that of Arensmen et al., (1996) which states that psychiatric disorders are less documented in attempters than completers and are reported to be more common in repeaters than first attempters.

Certain limitation of our study needs to be mentioned here. It was conducted on hospitalized cases and the cases were only from general medical ward. So this sample might have selection bias. Further exploration by a study on a sample drawn from community-based population is needed for generalizing the findings to the population of this region.

In summary our study had identified pain and physical disorders as significant risk factors for suicide attempt in addition to the other psychosocial conditions like unemployment, lack of education and exposure to stressful life events. The main implication of our findings is that the young patients of abdominal and pelvic pain attending medical clinics require careful assessment and detailed appraisal of their psychosocial conditions for assessing the risk of attempting suicide in such cases.

### REFERENCES

Arensmen, E. & Kerkhof, A.J.F.M. (1996) Causes of attempted suicide: a review of empirical studies 1963-1993. Suicide and Life threatening behaviour, 26,46-47

Badrinarayana, A. (1977) Suicide attempts in Gulbarga. Indian Journal of Psychiatry, 19(4), 69-70

Botsis, A.J., Plutckik, R., Kotler, M., et al. (1995) Parental loss and family violence as correlates of suicide and violent risk. Suicide & Life Threatening Behaviour, 121, 246-298

Chastang, F., Rioux, P., Dupont, I., Baranger, E., Kovess, V., & Zarifian, E.(1998) Risk factors associated with suicide attempt in young French people. Acta Psychiatrica Scandinavica, 98,474-479

Folkman, S., Lazarus, R.S., Pimley, S., & Novacek, J.C.,( 1987) Age difference in stress and coping process. Psychology and Aging , 2, 171-184

Jensen, M.P., Turner J.A., Romano, J.M., Karoly, P. (1991) Coping with chronic pain: a critical review of literature. Pain, 47, 249-283

Hawton, K., O'Grady, J., Osborn, M., & Cole, D. (1982) Adolescents who take overdoses their characteristics, problems, and contacts with helping agencies. British Journal of Psychiatry, 140, 118-123

Hintikka,J., Kontula,O.,Saarinen P., Tanskanen, A., Koskela, K., & Viinamaki, H. ( 1998) Debt and suicidal behaviour in Finnish general population. Acta Psychiatrica Scandinavica, 98, 493-496

Kirmayer, L.J., Malus, M., & Boothroyd, L.J. (1996) Suicide attempts among Inuit youths: a community survey of prevalence and risk factors. Acta Psychiatrica Scandinavica, 94, 8-17

Latha, K.S., Bhat, S.M. & D'Souza, P. (1996) Suicide attempters in a general hospital unit in India: their socio-demographic and clinical profile-emphasis on cross-cultural aspects. Acta Psychiatrica Scandinavica, 94,26-30

Lal, N. & Sethi, B.B. (1975) Demographic and socioeconomic variables in attempted suicide by poisoning. Indian Journal of Psychiatry, 17,100-107

Miles, C.P. (1977) Conditions predisposing to suicide: a review. Journal of Nervous and Mental diseases, 164,231-246

Narang, R. L., Mishra, B. P. & Mohan, N.(2000) Attempted suicide in Ludhiana. Indian Journal of Psychiatry, 42(1), 83-87

Paykel, E.S., Prusoff, B. & Myers, J.K. (1974) Suicide attempts and recent life events: a controlled comparison. Archives of General Psychiatry, 32,327-333

Ponnudurai, R., Jeykar, J. & Saraswathy, M.(1986) Attempted suicide in Madras. Indian Journal of Psychiatry, 28,59-62

Runeson, B.(1989) Mental disorder in youth suicide, DSMIII-R Axes I & II. Acta Psychiatrica Scandinavica, 79, 490-497.

Sathyavathi, K.(1971) Attempted suicide in psychiatric patients. Indian Journal of Psychiatry, 13(1), 37-46

Sathyavathi, K., MurtiRao, D.L.N.(1961) A study of Suicide in Bangalore. Trans All India Institute of mental health, 2, 1 Bangalore

Suominen, K., Henriksson, M., Suokas, J., Isometsa, E., Ostamo, A., Lonnqvist, J.(1996) Mental disorders and comorbidity in attempted suicide. Acta Psychiatrica Scandinavica, 94,234-240

Stenager, E.N. & Stenager, E. (2000) Physical illness and suicidal behaviour. In The International Handbook of Suicide and Attempted suicide, Hawton, K., &Heeringen, K.V. (eds), Ist edition, pp. 405-416,John Wiley and Sons.

Schmidtke, A., Bille-Brahe, U., DeLeo, D., Kerkhof, A., Bjerke, T., Crepet., P. et al.(1996)Attempted suicide in Europe, rates trends socio-demographic characteristics of suicide attempters during the period 1989-1992, Results of WHO/EURO Multicentric study on parasuicide. Acta Psychiatrica Scandinavica, 93,327-338

Singh, G.,Kaur,D & Kaur, H.(1983) Handbook of Presumptive life events scale, Agra, National Psychological Corporation.

Sudhirkumar, C.T. & Chandrasekaran, R.(2000) A study of psychosocial and clinical factors associated with adolescent suicide attempt. Indian Journal of Psychiatry, 42(3), 237-242.

Venkoba Rao, A. (1971) Suicide attempters in Madurai. Journal of Indian Medical Association, 57, 278-283

World Health Organisation (1992) The ICD -10 Classification of Mental and Behavioral disorders. Clinical descriptions and diagnostic guidelines, Geneva: WHO

World Health Organisation (1992) International Statistical Classification of diseases and related health problems 10th Revision vol 1,pp 859, WHO, Geneva

## References

[PDF_00461] Chastang F., Rioux P., Dupont I., Baranger E., Kovess V., Zarifian E. (1998). Risk factors associated with suicide attempt in young French people.. Acta Psychiatr Scand.

[PDF_00466] Folkman S., Lazarus R. S., Pimley S., Novacek J. (1987). Age differences in stress and coping processes.. Psychol Aging.

[PDF_00470] Hawton K., O'Grady J., Osborn M., Cole D. (1982). Adolescents who take overdoses: their characteristics, problems and contacts with helping agencies.. Br J Psychiatry.

[PDF_00473] Hintikka J., Kontula O., Saarinen P., Tanskanen A., Koskela K., Viinamäki H. (1998). Debt and suicidal behaviour in the Finnish general population.. Acta Psychiatr Scand.

[PDF_00468] Jensen M. P., Turner J. A., Romano J. M., Karoly P. (1991). Coping with chronic pain: a critical review of the literature.. Pain.

[PDF_00476] Kirmayer L. J., Malus M., Boothroyd L. J. (1996). Suicide attempts among Inuit youth: a community survey of prevalence and risk factors.. Acta Psychiatr Scand.

[PDF_00513] Kumar C. T., Chandrasekaran R. (2000). A study of psychosocial and clinical factors associated with adolescent suicide attempts.. Indian J Psychiatry.

[PDF_00479] Latha K. S., Bhat S. M., D'Souza P. (1996). Suicide attempters in a general hospital unit in India: their socio-demographic and clinical profile--emphasis on cross-cultural aspects.. Acta Psychiatr Scand.

[PDF_00485] Miles C. P. (1977). Conditions predisposing to suicide: a review.. J Nerv Ment Dis.

[PDF_00489] Paykel E. S., Prusoff B. A., Myers J. K. (1975). Suicide attempts and recent life events. A controlled comparison.. Arch Gen Psychiatry.

[PDF_00516] Rao A. V. (1971). Suicide attempters in Madurai.. J Indian Med Assoc.

[PDF_00494] Runeson B. (1989). Mental disorder in youth suicide. DSM-III-R Axes I and II.. Acta Psychiatr Scand.

[PDF_00506] Schmidtke A., Bille-Brahe U., DeLeo D., Kerkhof A., Bjerke T., Crepet P., Haring C., Hawton K., Lönnqvist J., Michel K. (1996). Attempted suicide in Europe: rates, trends and sociodemographic characteristics of suicide attempters during the period 1989-1992. Results of the WHO/EURO Multicentre Study on Parasuicide.. Acta Psychiatr Scand.

[PDF_00500] Suominen K., Henriksson M., Suokas J., Isometsä E., Ostamo A., Lönnqvist J. (1996). Mental disorders and comorbidity in attempted suicide.. Acta Psychiatr Scand.

